# Neural regulation of energy and bone homeostasis by the synaptic adhesion molecule Calsyntenin-3

**DOI:** 10.1038/s12276-020-0419-8

**Published:** 2020-05-07

**Authors:** Sung-Jin Kim, Yong Taek Jeong, Se Rok Jeong, Munsu Park, Hye Sun Go, Mi Young Kim, Je Kyung Seong, Ki Woo Kim, Jeong Taeg Seo, Chul Hoon Kim, Ji Hyun Lee, Seok Jun Moon

**Affiliations:** 1 Department of Oral Biology, BK21 PLUS Project, Yonsei University College of Dentistry, Yonsei-ro 50-1, Seodaemun-gu, Seoul, 03722 Korea; 2 Department of Pharmacology, Korea University College of Medicine, Seoul, 02841 Korea; 3 Department of Clinical Pharmacology and Therapeutics, College of Medicine, Kyung Hee University, Seoul, Korea; 4 Korea Mouse Phenotyping Center (KMPC), Seoul National University, 1 Gwanak-ro, Gwanak-gu, Seoul, 08826 Korea; 5 Department of Pharmacology, Yonsei University College of Medicine, Yonsei-ro 50-1, Seodaemun-gu, Seoul, 03722 Korea

**Keywords:** Homeostasis, Bone

## Abstract

Neuronal regulation of energy and bone metabolism is important for body homeostasis. Many studies have emphasized the importance of synaptic adhesion molecules in the formation of synapses, but their roles in physiology still await further characterization. Here, we found that the synaptic adhesion molecule Calsyntenin-3 (CLSTN3) regulates energy and bone homeostasis. *Clstn3* global knockout mice show reduced body mass with improved leptin sensitivity and increased energy expenditure compared to their wild-type littermates. In addition, *Clstn3* knockout mice show reduced marrow volume and cortical bone mass without alteration of trabecular bone microarchitecture. This reduced bone mass is not bone cell-autonomous because neither osteoblast- nor osteoclast-specific *Clstn3* knockout mice show bone defects; similarly, in vitro cultures of both *Clstn3* knockout osteoblasts and osteoclasts do not show any defects. These reduced body and bone mass phenotypes can be attributed instead to neuronal CLSTN3 because they are recapitulated by pan-neuronal but not sympathetic neuron-specific deletion of *Clstn3*. This study reveals novel physiological functions of neuronal *Clstn3* as a key regulator of energy and bone homeostasis.

## Bone metabolism: Regulation by brain protein

A protein that is highly expressed in brain cells plays a vital role in maintaining balanced energy expenditure and bone health. Recent research indicates that protein activity within brain cells can directly influence energy and bone metabolism. Disruption to these proteins may therefore be associated with obesity and osteoporosis. In experiments on mice, Seok Jun Moon at Yonsei University College of Dentistry in Seoul, South Korea and co-workers found that the protein calsyntenin-3 is expressed at high levels in brain cells. Mice lacking the protein had reduced body mass and growth rate, increased energy expenditure, and lower overall bone mass. This was true regardless of their diet, suggesting they were resistant to diet-induced obesity. The team believe calsyntenin-3 is one of several key brain-based regulators of energy and bone metabolism.

## Introduction

As common chronic diseases, respectively, associated with dysregulation of energy and skeletal homeostasis, obesity and osteoporosis are associated with significant morbidity and mortality. Thus, in an effort to ease the burden of these diseases, we need to prioritize improving our understanding of the mechanisms underlying energy and skeletal homeostasis. Given that the brain is a master regulator of peripheral homeostasis in general, it should not be surprising that the brain integrates various internal and external inputs to regulate energy metabolism^1,2^. More recently, however, evidence has accumulated that the brain also regulates bone homeostasis^3,4^.

Although several molecules and pathways involved in the central regulation of energy and skeletal homeostasis have been identified and characterized independently of one another, the brain’s regulation of bone and adipose tissue actually seems to be linked. For example, leptin, the fat-derived hormone that controls energy homeostasis and expenditure via hypothalamic neural circuits, is also an important regulator of bone homeostasis that acts at least in part by regulating sympathetic tone^5^. We expect that a better understanding of the mechanisms underlying the central coregulation of energy and skeletal homeostasis will accelerate the development of new interventions with dual therapeutic effects on both obesity and osteoporosis.

Synaptic adhesion molecules (SAMs) are proteins involved in the formation, differentiation, and plasticity of chemical synapses^6^. Recent evidence suggests that SAMs may be key players in the coregulation of bone and energy homeostasis. Genome-wide association studies of body mass index discovered many novel genes with unexpected functions, including SAMs such as *NRXN3*, *CADM1*, and *CADM2*^7,8^. Subsequent molecular studies demonstrated that *Cadm1* knockout results in reduced body and bone mass with improved leptin sensitivity and increased energy expenditure, suggesting that *Cadm1* simultaneously regulates both energy and bone homeostasis^9,10^.

Calsyntenin-3 (*Clstn3*) is a recently identified SAM in the cadherin superfamily. *Clstn3* is predominantly expressed postsynaptically in various brain regions and is required for presynapse differentiation mainly via its interaction with α-neurexin^11,12^. It is found in the cerebrospinal fluid of patients with Alzheimer’s disease (AD)^13^ and dystrophic neurites in AD brains^14^, suggesting that it may contribute to the pathogenesis of AD. Recently, Zeng et al.^15^ reported that *Clstn3b*, which shares the two C-terminal exons of *Clstn3*, regulates whole-body energy expenditure by controlling the sympathetic innervation of brown adipose tissues (BAT), but the precise physiologic function of *Clstn3* is still unclear.

Here, we sought to investigate the role that *Clstn3* plays in the regulation of energy and bone homeostasis. In contrast to *Clstn3b* global knockout mice, which show obesity due to reduced sympathetic innervation of BAT, *Clstn3* global knockout mice show reduced body mass due to improved leptin sensitivity and increased energy expenditure. In addition, these mice have reduced marrow volume and cortical bone mass without alteration of the trabecular bone architecture. Finally, we were able to recapitulate both the body mass and bone phenotypes via pan-neuron-specific but not sympathetic neuron-specific deletion of *Clstn3*. Together, these results identify *Clstn3* as a key regulator of energy and bone metabolism in the brain.

## Materials and methods

### Mice

All animal experiments were approved by the Animal Care Committee of Yonsei University College of Medicine. All mice were maintained under standard animal housing conditions (12-hour light-dark cycles with free access to food and water). Food was provided as a normal chow diet (#5053, PicoLab, St. Louis, MO) or high-fat diet (HFD; 60% kcal fat, # D12492, Research Diets, Brunswick, NJ). *Clstn3*^*tm1a*^ knockout first allele mice (strain C57BL/6) were purchased from the European Mouse Mutant Archive (C57BL/6N-*Clstn3*^*tm1a(EUCOMM)Hmgu*^). To obtain *Clstn3*^*tm1b*^ mice (i.e., a lacZ-tagged global knockout allele, *Clstn3*(−)), *Clstn3*^*tm1a*^ mice were crossed with transgenic *CMV-Cre* mice (stock no. 006054, The Jackson Laboratory), and then the Cre recombinase was removed by crossing with C57BL/6 wild-type mice. To obtain *Clstn3*^*tm1c*^ (i.e., the floxed allele, *Clstn3*^*fl*^), *Clstn3*^*tm1a*^ mice were crossed with transgenic *ACTB-FLP1* mice (stock no. 005703, The Jackson Laboratory), and then the FLP1 recombinase was removed by crossing with C57BL/6 wild-type mice. Tissue-specific conditional knockout mice were generated by crossing *Clstn3*^*fl/fl*^ with transgenic *Synapsin I*-*Cre* mice (*Syn I-Cre*; stock no. 003966, The Jackson Laboratory), *Dbh-Cre* (stock no. 032081-UCD, MMRRC), *Osteocalcin*-*Cre* (*Ocn-Cre*; stock no. 019509, The Jackson Laboratory), or *Lysozyme*-*Cre* (*LysM-Cre*; stock no. 004781, The Jackson Laboratory). Genotyping was performed by genomic PCR using genomic DNA extracted from mouse tails (MyTaq Extract-PCR kit, BIO-21127, Bioline, UK). The primer sequences for the transgenic *Cre* mice were as follows: *Cre* sense, 5’-TCCAATTTACTGACCGTACACCAA-3’*; Cre* antisense, 5’-CCTGATCCTGGCAATTTCGGCTA-3’. The primer sequences for genotyping the *Clstn3* alleles were as follows: shared sense, 5’-ACTTGATCAGTCCTCCTGCATCAG-3’; wild-type and *tm1c* antisense, 5’-CCTTCCTCCTACATAGTTGGCAGT-3’; *tm1a* antisense, 5’- CTGAAGTTCAGGGTCAGCCTGTAA-3’; and *tm1b* antisense, 5’-CCAAGATGGTGGCCAGGCTTAG-3’. Only male mice were used for all analyses.

### Histology

Epididymal white adipose tissue (WAT) and interscapular BAT were dissected from 17-week-old wild-type and *Clstn3*(−/−) littermates before being fixed in 10% neutral buffered formalin for 2 days. Then, the specimens were processed for paraffin embedding, cut into 4 μm sections, and subjected to H&E staining. Light microscopic images were obtained at ×200 magnification, and three randomly chosen WAT images were analyzed for area and number of adipocytes using ImageJ (National Institutes of Health, Bethesda, MD).

### Glucose and insulin tolerance tests

For glucose tolerance tests, 16-week-old mice were fasted for 18 h with water provided ad libitum. After fasted glucose levels were measured using blood samples collected from a tail nick with a glucometer (Ascensia Contour, Bayer HealthCare, Germany), glucose (1 g/kg) was intraperitoneally injected, and glucose levels were measured 15, 30, 60, 90, and 120 min after injection. For insulin tolerance tests, 17-week-old mice were fasted for 2 h with water provided ad libitum. After measuring basal glucose levels, insulin (0.9 U/kg) was intraperitoneally injected, and glucose levels were measured at the given time points after injection.

### Food intake and leptin sensitivity test

To measure food intake, 16-week-old wild-type and *Clstn3*(−/−) littermates were individually caged with normal chow and water provided *ad libitum*. After 3 days of acclimation, food intake was recorded daily at 9:00 and 18:00 over the course of 3 days and normalized to body weight. Leptin sensitivity was measured in 8-week-old wild-type and *Clstn3*(−/−) littermates by injecting recombinant murine leptin (1 mg/kg; from Dr. A.F. Parlow of the National Hormone and Peptide Program in Torrance, CA) intraperitoneally twice a day at 9:30 and 18:30 h for 3 days. Then, food intake was measured daily at 9:00.

### Measurement of serum leptin, norepinephrine (NE), osteocalcin (OCN), and the cross-linked C-telopeptide of type I collagen (CTX-I)

Blood samples were collected from 17-week-old mice at 14:00 with food and water provided *ad libitum*. Serum was separated by centrifuging and removing the blood clot 30 min after blood collection. We used ELISA kits according to the manufacturer’s instructions to measure leptin (#90040, Morinaga Institute of Biological Science, Yokohama, Japan), NE (BA-E-5200, Labor Dianostika Nord GmbH & Co., KG, Nordhorn, Germany), OCN (J64239, Alfa Aesar, Word Hill, MA), and CTX-I (AC-06F1, Immunodiagnostic systems, UK).

### Metabolic analysis

The metabolic rate was measured in 16-week-old wild-type and *Clstn3*(−/−) littermates fed normal chow. The mice were acclimated for 2 days individually in a combined indirect calorimetry system (CaloSys Calorimetry System, TSE Systems, Inc., Bad Homburg, Germany) with food and water. After acclimation, heat generation, O_2_ consumption, and CO_2_ production were measured for 48 h, and the metabolic rate was normalized using metabolic body weight (body weight^0.75^). During the measurement, locomotor activity was assessed by recording the number of infrared beam breaks caused by the animal’s ambulatory and fine movements.

### μCT analysis

After euthanizing the mice and dissecting their femurs, the femurs were fixed in 10% neutral buffered formalin for 2 days. The fixed femurs were then stored in 70% ethanol at 4 °C until μCT scanning. The femurs were scanned using SkyScan 1173 (Bruker, Kontich, Belgium) with an isotropic voxel size of 7.103 μm using settings of 90 kV, 88 μA, a 1.0 mm aluminum filter, and an X-ray detector with 2240 × 2240 pixels. The acquired images were reconstructed into cross-sectional images using NRecon (version 1.6.10, Bruker) and then analyzed with CTAn (version 1.16, Bruker). The regions of interest were defined as 0.5–1.7 mm proximal to the growth plate in the distal metaphysis and 0.4 mm in the midshaft region. A grayscale threshold value range of 62–255 was used for morphometric analysis. Three-dimensional volume-rendered images were acquired using CTVox (version 3.1.2, Bruker).

### Cell culture

Primary osteoblasts were obtained by enzyme digestion of the calvaria from 1–3-day-old wild-type and *Clstn3*(−/−) neonates with 0.1% collagenase type IV (#C5138, Sigma-Aldrich, St. Louis, MO) and 0.2% dispase II (#17105–041, Gibco, Waltham, MA). Cells were cultured in complete growth medium (α-MEM; #12571–063, Gibco, 10% fetal bovine serum; #10099–141, Gibco, and 1% penicillin/streptomycin; #15140–122, Gibco). When cell confluency reached 80–90%, the cells were trypsinized and seeded into either 12-well (0.5 × 10^5^ cells/well) or 6-well (1.3 × 10^5^ cells/well) plates. Differentiation was induced 2 days after seeding by the addition of 50 μg/ml L-ascorbic acid (A4544, Sigma-Aldrich) and 10 mM final concentration β-glycerophosphate (G9422, Sigma-Aldrich) to the complete growth medium.

For osteoclast differentiation, bone marrow-derived macrophages (BMMs) were isolated from 6-week-old wild-type and *Clstn3*(−/−) mouse femurs and tibias. After lysing the red blood cells with ACK lysing buffer (A1049201, Thermo Fisher Scientific, Waltham, MA), BMMs were seeded into petri dishes and cultured for 3 days in complete growth medium with 30 ng/ml recombinant murine M-CSF (#315–02, PeproTech, Rocky Hill, NJ). Then, the adherent M-CSF-dependent macrophages were scraped and seeded into 96-well plates at a concentration of 1.2 × 10^4^ cells per well. Differentiation was induced for 5 days via the addition of 30 ng/ml recombinant murine M-CSF and 20 ng/ml recombinant murine RANKL (462-TEC, R&D Systems, Minneapolis, MN) to the complete growth medium.

### Alkaline phosphatase, alizarin red, and TRAP staining

Alkaline phosphatase (ALP) staining was performed after 5 days of differentiation of primary osteoblasts using the BCIP/NBT Liquid Substrate System (B1911, Sigma-Aldrich). ALP activity was also measured using the Alkaline Phosphatase Yellow (pNPP) Liquid Substrate System for ELISA (P7998, Sigma-Aldrich) according to the manufacturer’s instructions. For alizarin red staining, cells were fixed after 2–3 weeks of differentiation in 10% neutral buffered formalin and stained with a 1.36% alizarin red S (A5533, Sigma-Aldrich) solution (pH 4.1–4.3) for 1 h in the dark. For quantification, alizarin red S was extracted by incubation in a 10% cetylpyridinium chloride (CPC; C0732, Sigma-Aldrich) solution for 1 h. The collected CPC was transferred to a 96-well plate (0.2 ml/well), and the absorbance was read at 570 nm using an ELISA reader. For TRAP staining, cells were fixed in a 4% paraformaldehyde solution in phosphate-buffered saline and stained for TRAP using a leukocyte acid phosphatase kit (#387, Sigma-Aldrich) according to the manufacturer’s protocol. TRAP-positive cells with three or more nuclei were counted.

### Conventional and quantitative real-time reverse transcription PCR

Using the RNeasy Mini Kit (Qiagen, Hilden, Germany) according to the manufacturer’s protocol, total RNA was isolated from the hypothalamus of male mice, primary osteoblasts before and 21 days after osteogenic differentiation, and primary BMMs before and 5 days after osteoclastogenic differentiation. Reverse transcription (RT) was performed using 2 μg of RNA and oligo(dT) primers with the RevertAid RT Kit (EP0441, Thermo Fisher Scientific) according to the manufacturer’s protocol. For conventional RT-PCR, cDNAs were amplified using the MyTaq Extract-PCR kit (BIO-21127, Bioline, London, UK), and the PCR products were visualized on a 1.5% agarose gel. The SensiFAST SYBR Hi-ROX kit (BIO-92020, Bioline) was used for quantitative real-time RT-PCR (qPCR). The qPCR results were normalized using the *Gapdh* or *Rn18s* housekeeping genes. The primer sequences used in this study are listed in Supplementary Table 1.

### Propranolol treatment

Propranolol (P0884, Sigma-Aldrich) was administered to wild-type and *Clstn3*(−/−) male littermates in their drinking water for 10 weeks, from age 8–18 weeks, at a concentration of 0.5 mg/ml (a dose previously reported to affect bone in mice with increased sympathetic tone^5,16^). The propranolol-laced water was changed three times per week, while untreated mice were supplied with normal drinking water.

### Bulk tissue RNA sequencing and analysis

Using the RNeasy Mini Kit (Qiagen, Hilden, Germany) according to the manufacturer’s protocol, total RNA was isolated from the hypothalamus and hippocampus of 16-week-old *Clstn3*(−/−) mice and their wild-type littermates (*n* = 3, each). Total RNA samples were converted into cDNA libraries using the TruSeq Stranded mRNA Sample Prep Kit (Illumina, San Francisco, CA). Subsequently, mRNA library sequencing was performed by Macrogen (Seoul, Korea) using an Illumina NovaSeq 6000 according to the manufacturer’s instructions. The resulting fastq data were mapped to mouse reference mm10 using the HISAT2 program to obtain a sample X gene matrix. The analysis was performed using the basic EdgeR pipeline^17^. To remove data that could be perceived as noise, we removed genes that appear in only one of 12 total samples. As a result, 14,846 genes from 15,841 genes in the raw data were used for downstream analysis. Finally, a negative binomial generalized log-linear model was applied to the read counts for each gene. The p-value for each gene was adjusted to the false discovery ratio (FDR) using the Benjamini and Hochberg method. Genes were considered to be differentially expressed if |log_2_FC| > 0 and FDR < 0.05 in KO samples versus WT samples.

### Single-cell data analysis

Basal-level single-cell RNA-seq data for the mouse hypothalamus were downloaded from NCBI GEO (accession GSE87544)^18^. The subsequent analysis was performed using the R package Seurat^19^ after selecting drops expressing more than 2000 genes and less than 9000 genes. A total of 1498 cells met these criteria and were used for further analysis. A log normalization followed by a principal component analysis (PCA) was performed on the top 2000 most variable genes. Using the top 17 PC values and a resolution value of 1, cell clusters were identified using a shared nearest neighbor (SNN) modularity optimization-based clustering algorithm. A total of 24 clusters were identified, and the clusters were visualized using t-distributed stochastic neighbor embedding (t-SNE). Then, marker genes were used to distinguish between neuronal and nonneuronal clusters: *Snap25*, *Syt1* (neuron); *Slc17a5* (glutamatergic neuron); *Slc32a1* (GABAergic neuron); *Olig1*, *Sox9*, *Cldn5*, *C1qa* (nonneuron). To confirm the distribution of the *Clstn3* and *Cadm1* genes, cells expressing each gene are highlighted in each color on the t-SNE plot.

### Statistical analyses

All data are expressed as the means ± s.e.m. Statistical differences among groups were analyzed using two-sample *t*-tests or one-way ANOVA with post-hoc Tukey tests (GraphPad Prism 5.0, GraphPad Software, San Diego, CA).

## Results

### *Clstn3* expression is increased in the hypothalamus of mice with diet-induced obesity

**Fig. 1:**
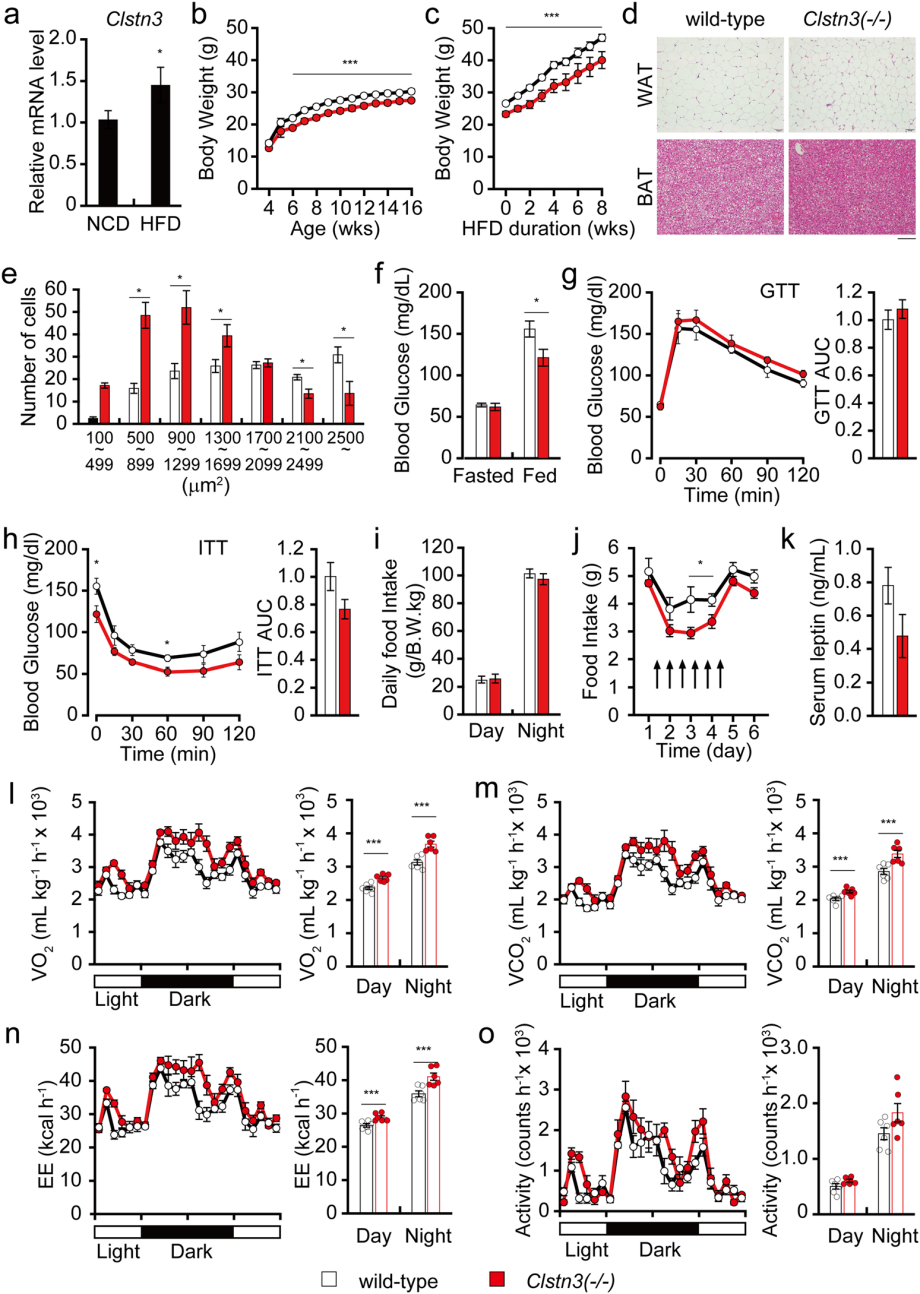
*Clstn3* knockout mice show reduced body mass with improved leptin sensitivity and increased energy expenditure. **a** qPCR analysis of *Clstn*3 in the hypothalamus of normal chow (NCD) or HFD-fed mice (n = 12). **b** Body weight of mice fed a NCD (n = 7 for wild-type and *n* = 8 for KO mice). **c** Body weight of mice fed a HFD from 8 weeks of age (*n* = 6 for wild-type and *n* = 4 for KO mice). **d** Representative H&E staining of epididymal WAT and BAT of 17-week-old mice. Scale bar, 0.1 mm. **e** Quantification of adipocyte size distribution in WAT (*n* = 7 for wild-type and *n* = 8 for KO mice). **f** Fed or fasted blood glucose levels in 16-week-old male mice (*n* = 5 for wild-type and *n* = 7 for KO mice). **g** Glucose tolerance test and resulting AUC in 16-week-old male mice (*n* = 5 for wild-type and *n* = 7 for KO mice). **h** Insulin tolerance test and AUC in 17-week-old male mice (*n* = 5 for wild-type and n = 7 for KO mice). **i** Normalized daily food intake of 16-week-old male mice on NCD (*n* = 7 for wild-type and *n* = 8 for KO mice). **j** Daily food intake in 16-week-old mice during leptin challenges (*n* = 6 for wild-type and *n* = 7 for KO mice). **k** Serum leptin levels in 17-week-old mice (*n* = 7 for wild-type and *n* = 6 for KO mice). **l**–**o** Metabolic analysis of 16-week-old mice (*n* = 6). **l** O_2_ consumption, **m** CO_2_ consumption, **n** energy expenditure, and **o** locomotor activity. Data are presented as the means ± s.e.m. Two-sample *t*-tests were performed for statistical analysis. *P < 0.05, ***P < 0.001.

Given that increased expression of several SAMs is associated with body mass index (BMI), we asked whether *Clstn3* expression is associated with BMI in a rodent animal model. We prepared mRNA from the hypothalamus of wild-type mice fed normal chow or HFD for eight weeks and performed qPCR. We found that *Clstn3* expression was significantly increased in the hypothalamus of HFD-fed mice (Fig. 1a). This is consistent with a recently published comparative transcriptome analysis of the hypothalamus from normal chow and HFD-fed mice^20^. Several SAMs showed increased expression in the hypothalamus of HFD-fed mice compared with normal chow-fed mice (Supplementary Table 2). These include the previously reported *Cadm1* as well as *Clstn3*. The increased hypothalamic expression of *Clstn3* in HFD-fed mice suggests that the altered expression of *Clstn3* induced by HFD may contribute to the regulation of body weight.

### *Clstn3* knockout mice show reduced body mass with improved leptin sensitivity and increased energy expenditure

To measure the contribution of *Clstn3* to the regulation of body weight, we generated global *Clstn3* knockout mice (*Clstn3*(−/−) or *Clstn3*^*tm1b/tm1b*^; Supplementary Fig. 1a). Since *Clstn3b*, which shares several exons with *Clstn3*, also regulates body weight, we confirmed that *Clstn3b* expression was unaltered in the hypothalamus of C*lstn3*(−/−) mice (Supplementary Fig. 1b). We monitored the body weight of *Clstn3*(−/−) and wild-type littermates fed a normal chow diet over 16 weeks. *Clstn3*(−/−) mice showed reduced body weight compared to wild-type littermates starting from 6 weeks of age (Fig. 1b). To determine whether this reduced body weight is associated with adipose tissue, we compared histological sections from epididymal WAT and supraclavicular BAT of *Clstn3*(−/−) and wild-type littermates (Fig. 1d). *Clstn3*(−/−) mice show reduced adiposity in WAT and BAT; *Clstn3*(−/−) fat cells show reduced diameter, indicating reduced fat content (Fig. 1d, e). To further investigate the role that CLSTN3 plays in adiposity and body weight during obesogenic conditions, we challenged *Clstn3*(−/−) mice with a HFD from 8 weeks of age for eight weeks. We observed that these mice showed significantly less weight gain than their wild-type littermates, indicating resistance to diet-induced obesity (Fig. 1c).

We performed insulin and glucose tolerance tests and found that *Clstn3*(−/−) mice show lower glucose levels in the random-fed state and upon insulin tolerance tests, suggesting an improvement in systemic insulin sensitivity (Fig. 1f–h). While their food intake remained normal (Fig. 1i), we found that intraperitoneal administration of leptin induced greater suppression of food intake in *Clstn3*(−/−) mice than in their wild-type littermates, suggesting that leptin sensitivity is enhanced in *Clstn3*(−/−) mice (Fig. 1j). Consistent with improved leptin sensitivity, *Clstn3*(−/−) mice tended to have lower serum leptin levels, although the difference was not statistically significant (Fig. 1k). Since *Clstn3*(−/−) mice seem to show increased insulin and leptin sensitivity, we next measured their rate of energy expenditure. *Clstn3*(−/−) mice show higher O_2_ consumption, CO_2_ production, and energy expenditure than their wild-type littermates (Fig. 1l–n), despite similar levels of physical activity (Fig. 1o). Together, these data strongly indicate that the lean phenotype of *Clstn3*(−/−) mice is, at least in part, due to improved leptin sensitivity and increased energy expenditure.

### *Clstn3* knockout mice show reduced bone mass

**Fig. 2:**
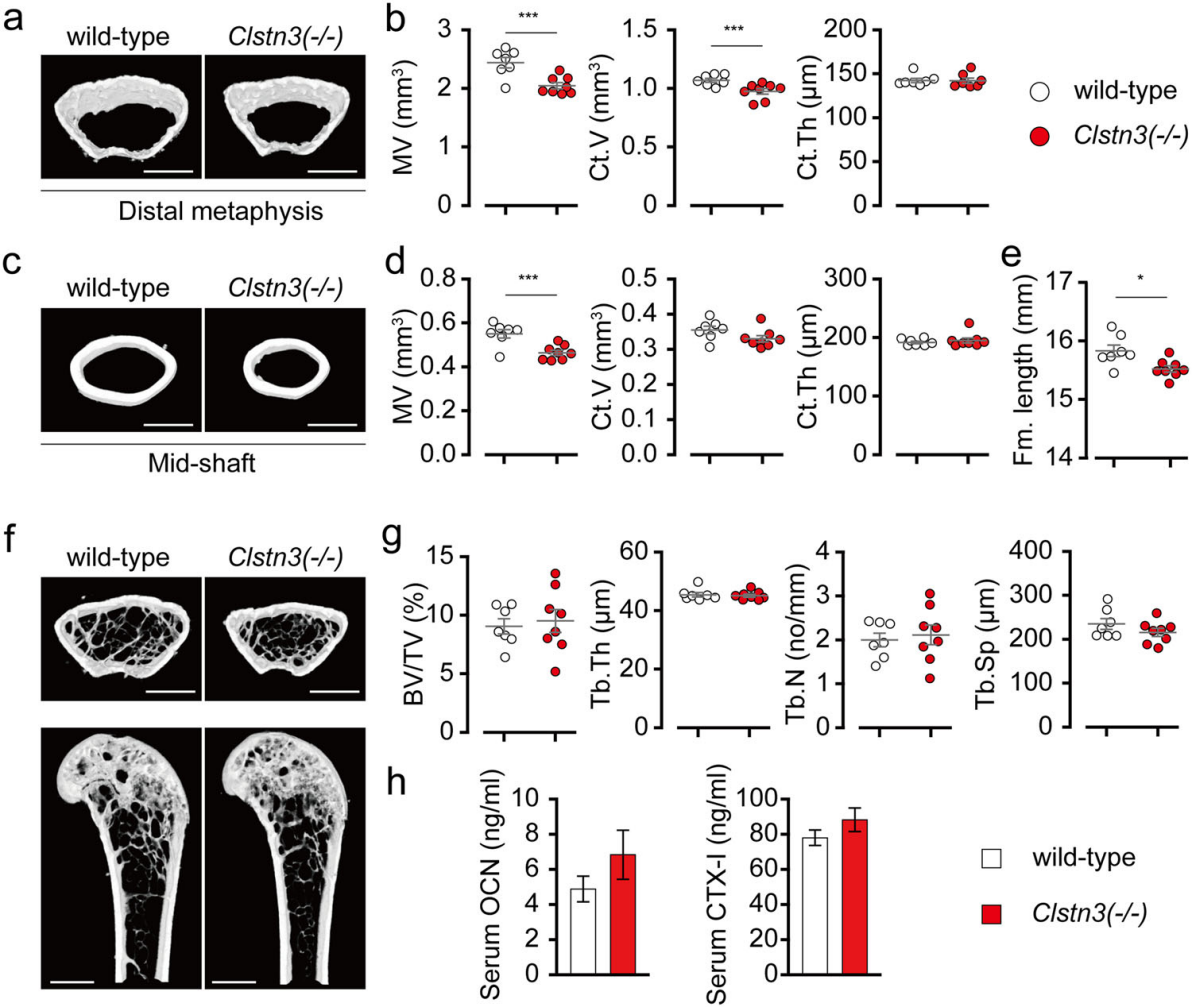
*Clstn3* knockout mice show reduced bone mass. **a**–**d** Femoral cortical bone analysis in 17-week-old male mice. Representative μCT images of cortical bone in the **a** distal metaphysis and **c** midshaft. Scale bar, 1 mm. Quantification of marrow volume (MV), cortical bone volume (Ct.V), and cortical bone thickness (Ct.Th) in the **b** distal metaphysis and **d** midshaft of femurs. **e** Average femur length in 17-week-old male mice. **f**, **g** Femoral trabecular bone analysis in 17-week-old male mice. **f** Representative μCT images of coronal (top) and longitudinal sections (bottom). Scale bar, 1 mm. **g** Quantification of the trabecular bone volume fraction (BV/TV), trabecular number (Tb.N), trabecular thickness (Tb.Th), and trabecular separation (Tb.Sp). **h** Serum levels of bone turnover markers in 17-week-old male mice (*n* = 7 for serum OCN, *n* = 7 and 8 for serum CTX-I levels of wild-type and KO mice, respectively). Data are presented as the means ± s.e.m. Two-sample *t*-tests were performed for statistical analysis. *P < 0.05, ***P < 0.001.

Since leptin signaling affects bone metabolism both centrally and peripherally^21^, we next asked whether CLSTN3 also regulates bone metabolism. Using μCT analysis, we found that marrow volume and cortical bone mass were significantly lower in *Clstn3*(−/−) mice than in littermate controls at the distal metaphysis and midshaft regions of the femur without any significant change in cortical bone thickness (Fig. 2a–d). Furthermore, femur length was significantly reduced in *Clstn3*(−/−) mice compared to littermate controls, suggesting that a reduced growth rate contributes at least in part to the reduced body mass of *Clstn3*(−/−) mice (Fig. 2e). The trabecular bone in the distal metaphysis, however, was not significantly different in volume fraction, thickness, number, or separation (Fig. 2f, g). In addition, serum levels of the bone formation marker OCN and the bone resorption marker CTX-I were similar in *Clstn3*(−/−) mice and littermate controls (Fig. 2h). These data indicate that CLSTN3 positively regulates bone mass without altering the microarchitecture of trabecular bone.

### The reduced bone mass in *Clstn3* knockout mice is not bone cell-autonomous

**Fig. 3:**
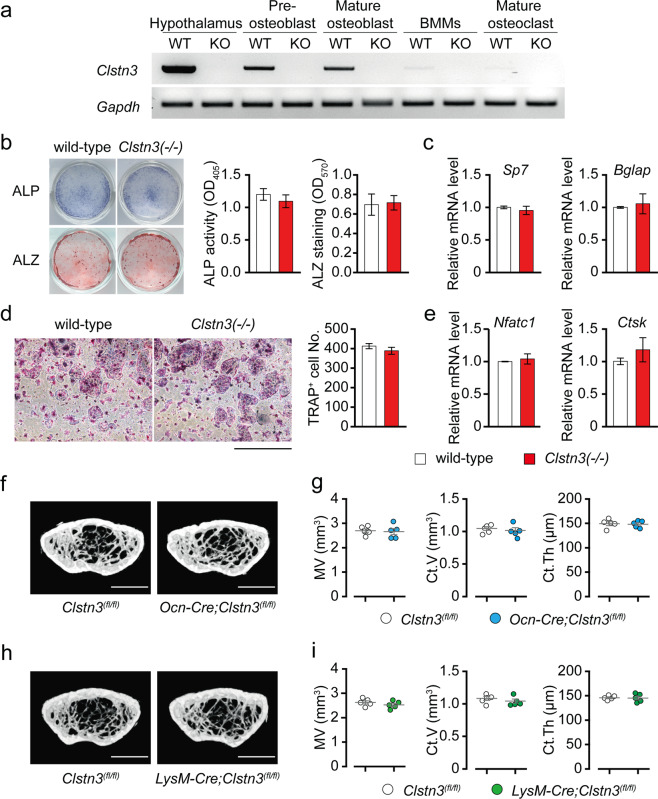
Reduced bone mass in *Clstn3* knockout mice is not bone cell autonomous. **a** Representative gel images of RT-PCR of *Clstn3* and *Gapdh* (loading control) in the indicated tissue or cells. **b** Representative images of ALP and ALZ staining (left) and a quantification of ALP activity and ALZ staining (right) (*n* = 4 and 6 for ALP activity and ALZ staining, respectively). **c** qPCR results for *Sp7* and *Bglap* in calvarial osteoblasts after 21 days of osteogenic differentiation (*n* = 4). **d** Representative images of TRAP staining of BMM cells (left) and quantification of multinucleated TRAP-positive cells (right) after 5 days of osteoclast differentiation in the presence of RANKL and M-CSF (*n* = 6). Scale bar, 1 mm. **e** qPCR results of *Nfatc1* and *Ctsk* in BMM cells after 5 days of osteoclast differentiation (*n* = 4). **f**–**i** μCT analysis of 16-week-old osteoblast-specific *Clstn3* knockout mice (*Ocn-Cre;Clstn3*^*fl/fl*^) and osteoclast-specific *Clstn3* knockout mice (*LysM-Cre;Clstn3*^*fl/fl*^). **f**, **h** Representative images of coronal sections in the distal femur metaphysis, and **g**, **i** a quantification of marrow volume (MV), cortical bone volume (Ct.V), and cortical bone thickness (Ct.Th). Scale bar, 1 mm. Data are presented as the means ± s.e.m. Two-sample *t*-tests were performed for statistical analysis.

To better understand how CLSTN3 regulates bone mass, we analyzed the expression of *Clstn3* in the hypothalamus, preosteoblasts, mature osteoblasts, BMMs, and mature osteoclast cells by RT-PCR. First, we confirmed the specificity of the *Clstn3* signal and that *Clstn3*(−/−) is a null allele by confirming that we could not detect any *Clstn3* transcripts in *Clstn3*(−/−) cells and tissues (Fig. 3a). Consistent with our previous results, the hypothalamus, a pivotal brain region for regulating energy homeostasis and bone mass, shows the highest expression of *Clstn3* mRNA (Fig. 3a). Although they show much lower levels than the hypothalamus, preosteoblasts and mature osteoblasts do express some *Clstn3*. The expression of *Clstn3* in BMMs and mature osteoclasts, however, is almost undetectable.

Because we detected *Clstn3* transcripts during osteoblast and osteoclast differentiation, we next asked whether the role of *Clstn3* in osteoblast or osteoclast differentiation is cell-autonomous. When we cultured primary osteoblasts in vitro, we found no difference in osteoblast differentiation between wild-type and *Clstn3*(−/−) osteoblasts, as demonstrated by ALP activity, ALZ staining, and qPCR of osteoblastic marker genes such as *Sp7* and *Bglap* (Fig. 3b, c). We also found that RANKL-induced osteoclast differentiation in vitro is similar in wild-type and *Clstn3*(−/−) BMMs via a TRAP assay and qPCR of osteoclast marker genes such as *Nfatc1* and *Ctsk* (Fig. 3d, e). To further explore the role of *Clstn3* in bone homeostasis and to determine whether it is cell-autonomous, we generated osteoblast-specific and osteoclast-specific *Clstn3* knockout mice using floxed *Clstn3* allele mice (*Clstn3*^*fl/fl*^ or *Clstn3*^*tm1c*^*/*^*tm1c*^) and *Ocn-Cre* and *LysM-Cre*, respectively. We found that osteoblast- and osteoclast-specific *Clstn3* knockout mice do not recapitulate the bone phenotype of global *Clstn3* knockout mice, suggesting that *Clstn3* regulates bone mass in a cell nonautonomous manner (Fig. 3f–i; Supplementary Fig. 2a, b).

### Neural-derived *Clstn3* regulates bone mass and body mass

**Fig. 4:**
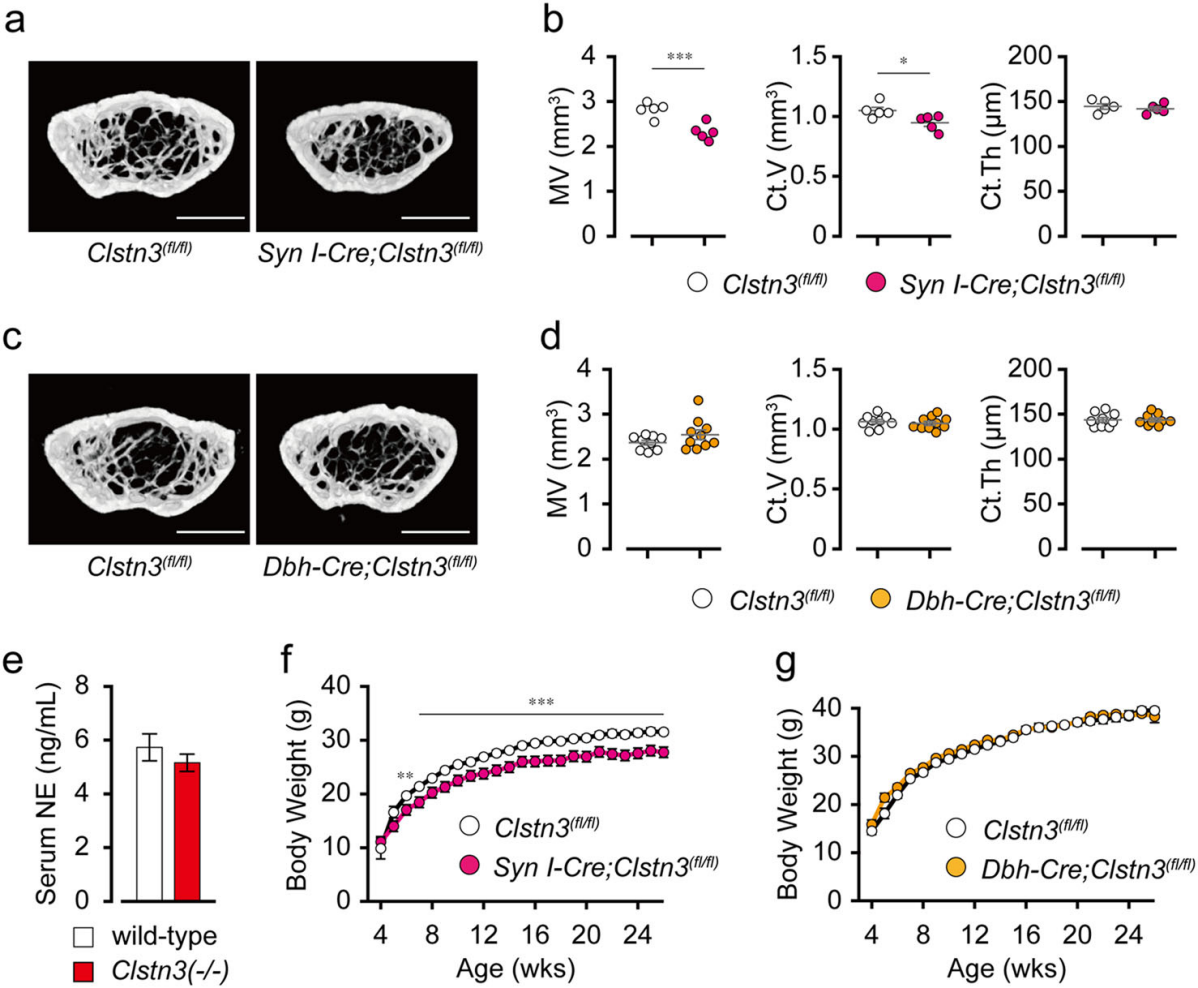
Neural-derived *Clstn3* regulates bone mass and body mass. **a**–**d** A μCT analysis of 16-week-old pan-neuronal *Clstn3* knockout mice (*Syn I-Cre;Clstn3*^*fl/fl*^) and sympathetic neuron-specific *Clstn3* knockout mice (*Dbh-Cre;Clstn3*^*fl/fl*^). **a**, **c** Representative images of coronal sections in the distal femur metaphysis, and **b**, **d** a quantification of marrow volume (MV), cortical bone volume (Ct.V), and cortical bone thickness (Ct.Th). Scale bar, 1 mm. **e** Serum NE level (*n* = 7 for wild-type and *n* = 8 for KO mice). **f**, **g** Body weight of **f** pan-neuronal *Clstn3* knockout mice, **g** sympathetic neuron-specific *Clstn3* knockout mice, and their floxed littermates (*n* = 8 and 9 mice for *Syn I-Cre;Clstn3*^*fl/fl*^ and their control, *n* = 10 and 7 mice for *Dbh-Cre;Clstn3*^*fl/fl*^ and their control, respectively). Data are presented as the means ± s.e.m. Two-sample *t*-tests were performed for statistical analysis. *P < 0.05, **P < 0.01, ***P < 0.001.

Since *Clstn3* is expressed primarily in neuronal tissue, we generated pan-neuron *Clstn3* knockout mice using *Syn I*-*Cre*. We found that neuron-specific *Clstn3* knockout mice recapitulated the bone phenotype of *Clstn3*(−/−) mice; they showed significantly lower marrow volume without altering the trabecular bone volume fraction (Fig. 4a, b and Supplementary Fig. 2c). We also found that pan-neuron-specific *Clstn3* knockout mice recapitulated the reduced body mass of *Clstn3*(−/−) mice (Fig. 4f). Because the alteration of sympathetic tone is the most well-known mechanism of central regulation of bone mass and peripheral metabolism^5^, we next generated sympathetic neuron-specific *Clstn3* knockout mice using *Dbh-Cre*. These mice, however, showed normal bone mass and body weight compared to littermate controls (Fig. 4c, d, g and Supplementary Fig. 2d). Serum NE levels were similar between *Clstn3*(−/−) knockout mice and littermate controls (Fig. 4e), and treatment with the nonselective beta-adrenergic receptor antagonist propranolol had no effect on any of the *Clstn3*(−/−) knockout mouse phenotypes (Supplementary Fig. 3). These data suggest that the regulation of bone mass and body weight by neural *Clstn3* is independent of sympathetic tone.

### *Clstn3* and *Cadm1* are coexpressed in the same population of hypothalamic neurons

**Fig. 5:**
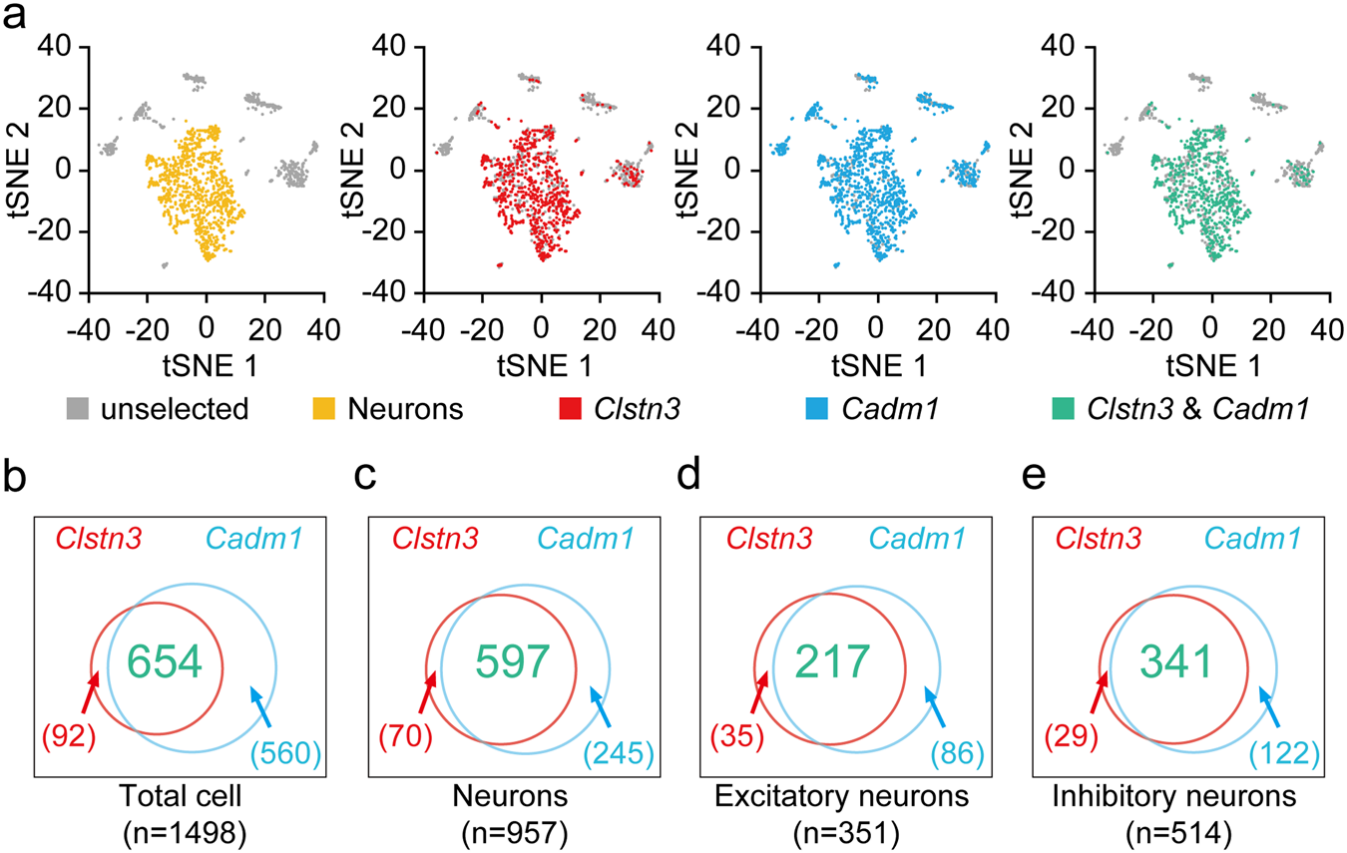
Expression of *Clstn3* and *Cadm1* in the hypothalamus. **a** t-SNE visualization of single cells identified by performing an unsupervised clustering analysis on published data. Yellow dots represent neurons, red dots are cells expressing *Clstn3*, blue dots are cells expressing *Cadm1*, and green dots are cells expressing *Clstn3* and *Cadm1* together. Gray dots indicate unselected cells. **b**–**e** Venn diagram for cells expressing *Clstn3* and *Cadm1*. The number of cells expressing only *Clstn3* is shown in red, the number of cells expressing only *Cadm1* is shown in blue, and the number of cells expressing both *Clstn3* and *Cadm1* genes is shown in green. **b** Venn diagram for *Clstn3-* and *Cadm1*-expressing cells in the total population. **c** Venn diagram of *Clstn3-* and *Cadm1*-expressing cells in neuronal cell populations. **d** Venn diagram of *Clstn3-* and *Cadm1*-expressing cells in the populations expressing the excitatory neuronal marker gene *Slc17a6*. **e** Venn diagram of *Clstn3-* and *Cadm1-*expressing cells in populations expressing the inhibitory neuronal marker gene *Slc32a1*. The numbers of cells in each group are indicated.

The metabolic and bone phenotypes of *Clstn3* knockout mice are markedly similar to those of *Cadm1* knockout mice^9,10^, suggesting that both SAMs may share a common signaling pathway in the regulation of energy and bone homeostasis. Since *Cadm1* functions in multiple brain regions including the hypothalamus^9^ to regulate body weight and energy homeostasis, we examined the expression patterns of *Clstn3* and *Cadm1* in the hypothalamus using a recently published single-cell RNA sequencing dataset (GSE87544). We found that while *Cadm1* is broadly expressed in neuronal and nonneuronal cells, *Clstn3* is primarily expressed in neuronal cells (Fig. 5a). *Clstn3* and *Cadm1* were coexpressed in 87.6% of *Clstn3*-positive cells and in 53.9% of *Cadm1*-positive cells among hypothalamic cells (Fig. 5b). Since the phenotypes of both *Clstn3* and *Cadm1* global knockout mice are recapitulated in neuron-specific knockout mice^9^, we focused on neuronal cells for our further analyses. When confined to neuronal cells, *Clstn3* and *Cadm1* were coexpressed in 89.5% of *Clstn3*-positive cells and in 70.9% of *Cadm1*-positive cells (Fig. 5c). Coexpression of *Clstn3* and *Cadm1* is not biased to either excitatory neurons (*Slc17a6*-positive neurons) or inhibitory neurons (*Slc32a1*-positive neurons) (Fig. 5d, e).

Next, we performed RNA sequencing in the hypothalamus and hippocampus of wild-type and *Clstn3* knockout littermates to gain molecular insight into what is mediating the metabolic and/or bone phenotypes. We did not find any significant difference in *Cadm1* or *Cadm2* mRNA expression in *Clstn3* knockout mice, indicating that deletion of *Clstn3* does not affect *Cadm1* and *Cadm2* expression at the transcript level (Supplementary Fig. 4a). Further analysis of differentially expressed genes revealed very few genes that are altered in *Clstn3* knockout mice. The most differentially expressed genes are *Sgk1*, which is upregulated in the hypothalamus and reported to mediate glucocorticoid-increased adiposity^22^, and *Mapt*, which is downregulated in the hippocampus and associated with autism spectrum disorder (Supplementary Fig. 4b–e)^23^.

## Discussion

Although it is now widely accepted that the brain provides central regulation of energy and bone metabolism, the precise mechanisms remain largely unknown. Here, we implicate neural *Clstn3* in the simultaneous regulation of energy and bone homeostasis. Global deletion of *Clstn3* reduces body mass while improving leptin sensitivity, increasing energy expenditure, and reducing bone mass and femur length without altering the microarchitecture of trabecular bone. All of these phenotypes are recapitulated by pan-neuronal deletion of *Clstn3*. Together, these data reveal previously unknown physiological functions for neuronal *Clstn3* as a key regulator of energy and bone homeostasis.

Currently, the best-known mechanism of central regulation of bone metabolism is a leptin-dependent alteration of sympathetic activity^5^. Leptin inhibits the synthesis and release of brainstem-derived serotonin, thereby increasing sympathetic tone to reduce bone mass^3,24^. Our results, however, suggest that the positive regulation of bone mass by neuronal *Clstn3* is unrelated to sympathetic activity. We found that sympathetic neuron-specific deletion of *Clstn3* neither affected bone mass (Fig. 4c, d) and that global deletion of *Clstn3* did not alter serum NE levels (Fig. 4e). We found that pharmacological blockade of sympathetic tone fails to rescue the global *Clstn3*(−/−) phenotype (Supplementary Fig. 3). Finally, although mouse models with altered sympathetic tone show changes in trabecular bone^5,25^, we found that *Clstn3* knockout mice show normal trabecular bone volume with reduced marrow volume and femur length. Since reduced sensory nerve innervation without any change in sympathetic innervation results in low bone mass^26^, it is intriguing to speculate that sensory nerve innervation or its synaptic transmission is altered in the bones of *Clstn3* knockout mice.

A recent study reported the function of a previously unidentified form of *Clstn3*, called *Clstn3b*, in energy homeostasis. *Clstn3b* consists of three exons, with the first exon residing in an intron of *Clstn3* and the last two exons shared with *Clstn3*. Interestingly, *Clstn3b* regulates energy homeostasis by controlling sympathetic innervation of BAT and thermogenesis in a direction opposite to that of *Clstn3*; *Clstn3b* knockout mice are obese and have increased blood glucose levels compared with wild-type littermates, whereas mice overexpressing *Clstn3b* in BAT are lean and resistant to diet-induced obesity. It is intriguing that a single genetic locus has evolved to produce different transcripts that control energy homeostasis in opposite directions.

The similarities in function and coexpression of *Clstn3* and *Cadm1* suggest that they share a common signaling pathway in the regulation of energy and bone homeostasis. Loss of *Sgk1* in hypothalamic POMC neurons was recently reported to lead to obesity with decreased energy expenditure, while overexpression of *Sgk1* in hypothalamic POMC neurons causes a lean phenotype with increased energy expenditure^22^. Therefore, *Sgk1* may be a downstream signaling mediator of *Clstn3* in its regulation of energy homeostasis. Future studies will be necessary to verify the genetic interactions between *Sgk1*, *Clstn3*, and *Cadm1*.

In conclusion, we have revealed novel physiological functions of *Clstn3* in regulating energy homeostasis by improving leptin sensitivity and increasing energy expenditure and in regulating bone mass independent of any alteration in sympathetic tone.

## Supplementary information

Supplementary figures and tables

## References

[1] Waterson MJ, Horvath TL (2015). Neuronal regulation of energy homeostasis: beyond the hypothalamus and feeding. Cell Metab..

[2] Roh E, Song DK, Kim MS (2016). Emerging role of the brain in the homeostatic regulation of energy and glucose metabolism. Exp. Mol. Med..

[3] Karsenty G, Oury F (2010). The central regulation of bone mass, the first link between bone remodeling and energy metabolism. J. Clin. Endocrinol. Metab..

[4] Huang S (2019). Neural regulation of bone remodeling: Identifying novel neural molecules and pathways between brain and bone. J. Cell Physiol..

[5] Takeda S (2002). Leptin regulates bone formation via the sympathetic nervous system. Cell.

[6] Washbourne P (2004). Cell adhesion molecules in synapse formation. J. Neurosci..

[7] Locke AE (2015). Genetic studies of body mass index yield new insights for obesity biology. Nature.

[8] Speliotes EK (2010). Association analyses of 249,796 individuals reveal 18 new loci associated with body mass index. Nat. Genet.

[9] Rathjen T (2017). Regulation of body weight and energy homeostasis by neuronal cell adhesion molecule 1. Nat. Neurosci..

[10] Yan X, Kononenko NL, Bruel A, Thomsen JS, Poy MN (2018). Neuronal cell adhesion molecule 1 regulates leptin sensitivity and bone mass. Calcif. Tissue Int..

[11] Pettem KL (2013). The specific alpha-neurexin interactor calsyntenin-3 promotes excitatory and inhibitory synapse development. Neuron.

[12] Um JW (2014). Calsyntenins function as synaptogenic adhesion molecules in concert with neurexins. Cell Rep..

[13] Ringman JM (2012). Proteomic changes in cerebrospinal fluid of presymptomatic and affected persons carrying familial Alzheimer disease mutations. Arch. Neurol..

[14] Uchida Y, Gomi F, Murayama S, Takahashi H (2013). Calsyntenin-3 C-terminal fragment accumulates in dystrophic neurites surrounding abeta plaques in tg2576 mouse and Alzheimer disease brains: its neurotoxic role in mediating dystrophic neurite formation. Am. J. Pathol..

[15] Zeng X (2019). Innervation of thermogenic adipose tissue via a calsyntenin 3beta-S100b axis. Nature.

[16] Motyl KJ (2013). Altered thermogenesis and impaired bone remodeling in Misty mice. J. Bone Min. Res..

[17] Robinson MD, McCarthy DJ, Smyth GK (2010). edgeR: a Bioconductor packagefor differential expression analysis of digital gene expression data. Bioinformatics.

[18] Chen R, Wu X, Jiang L, Zhang Y (2017). Single-cell RNA-Seq reveals hypothalamic cell diversity. Cell Rep..

[19] Satija R, Farrell JA, Gennert D, Schier AF, Regev A (2015). Spatial reconstruction of single-cell gene expression data. Nat. Biotechnol..

[20] Vagena E (2019). A high-fat diet promotes depression-like behavior in mice by suppressing hypothalamic PKA signaling. Transl. Psychiatry.

[21] Upadhyay J, Farr OM, Mantzoros CS (2015). The role of leptin in regulating bone metabolism. Metabolism.

[22] Deng Y (2018). SGK1/FOXO3 signaling in hypothalamic POMC neurons mediates glucocorticoid-increased adiposity. Diabetes.

[23] Abraham JR (2019). Proteomic investigations of autism brain identify known and novel pathogenetic processes. Sci. Rep..

[24] Yadav VK (2009). A serotonin-dependent mechanism explains the leptin regulation of bone mass, appetite, and energy expenditure. Cell.

[25] Elefteriou F, Campbell P, Ma Y (2014). Control of bone remodeling by the peripheral sympathetic nervous system. Calcif. Tissue Int..

[26] Fukuda T (2013). Sema3A regulates bone-mass accrual through sensory innervations. Nature.

