# Supplementary material for: Neural regulation of energy and bone homeostasis by the synaptic adhesion molecule Calsyntenin-3
Source: Exp Mol Med. 2020 May 7;52(5):793–803. doi: 10.1038/s12276-020-0419-8 (PMC7272401; doi:10.1038/s12276-020-0419-8)
Supplement: Supplementary file 1 — Supplementary figures and tables [file 12276_2020_419_MOESM1_ESM.pdf]

Supplementary Information

Neural regulation of energy and bone homeostasis by the synaptic adhesion molecule Calsyntenin-3

Sung-Jin Kim, Yong Taek Jeong, Se Rok Jeong, Munsu Park, Hye Sun Go, Mi Young Kim, Je Kyung Seong, Ki Woo Kim, Jeong Taeg Seo, Chul Hoon Kim, Ji Hyun Lee, and Seok Jun Moon

Supplementary Figure 1

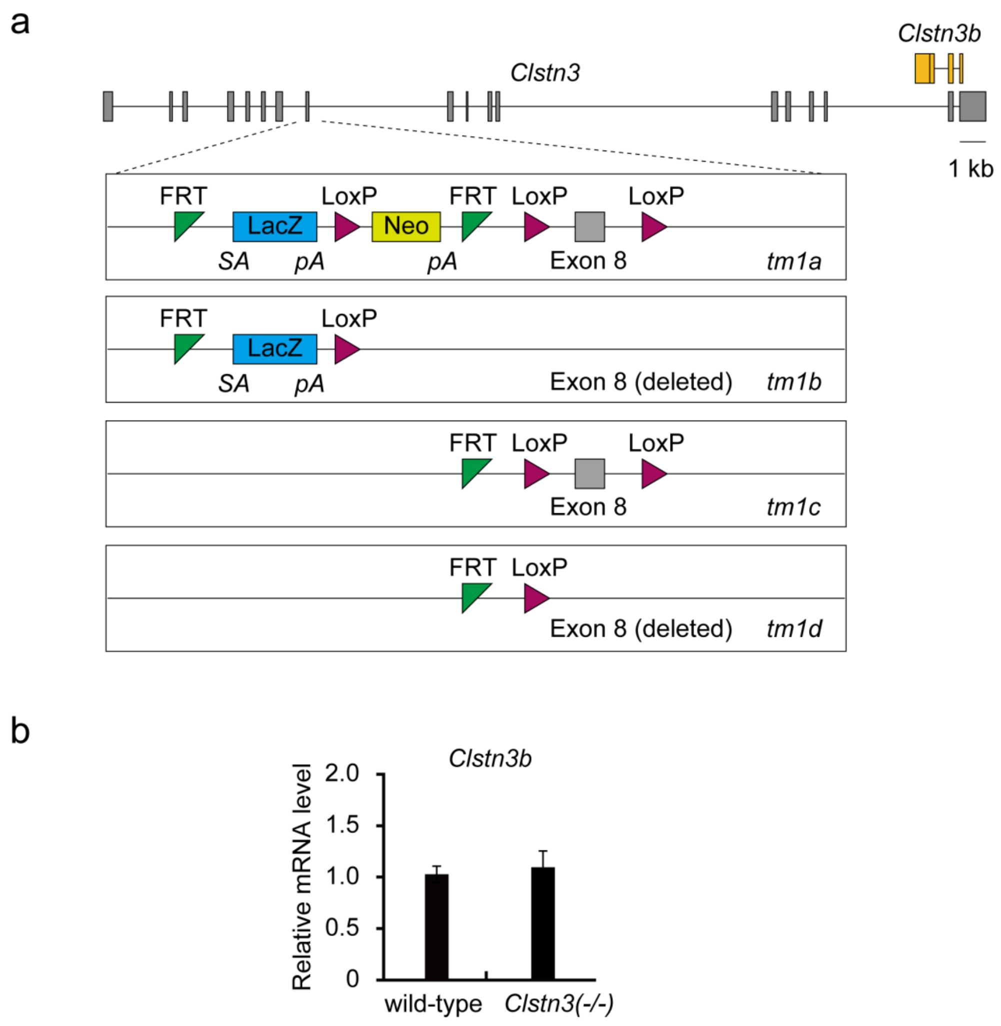

Supplementary Fig. 1 *Clstn3* locus. **a** *Clstn3* allele schematic. **b** qPCR analysis of *Clstn3b* transcripts in BAT of wild-type and *Clstn3*(-/-) mice (n=10). Two-sample *t*-tests were performed for statistical analysis.

Supplementary Figure 2

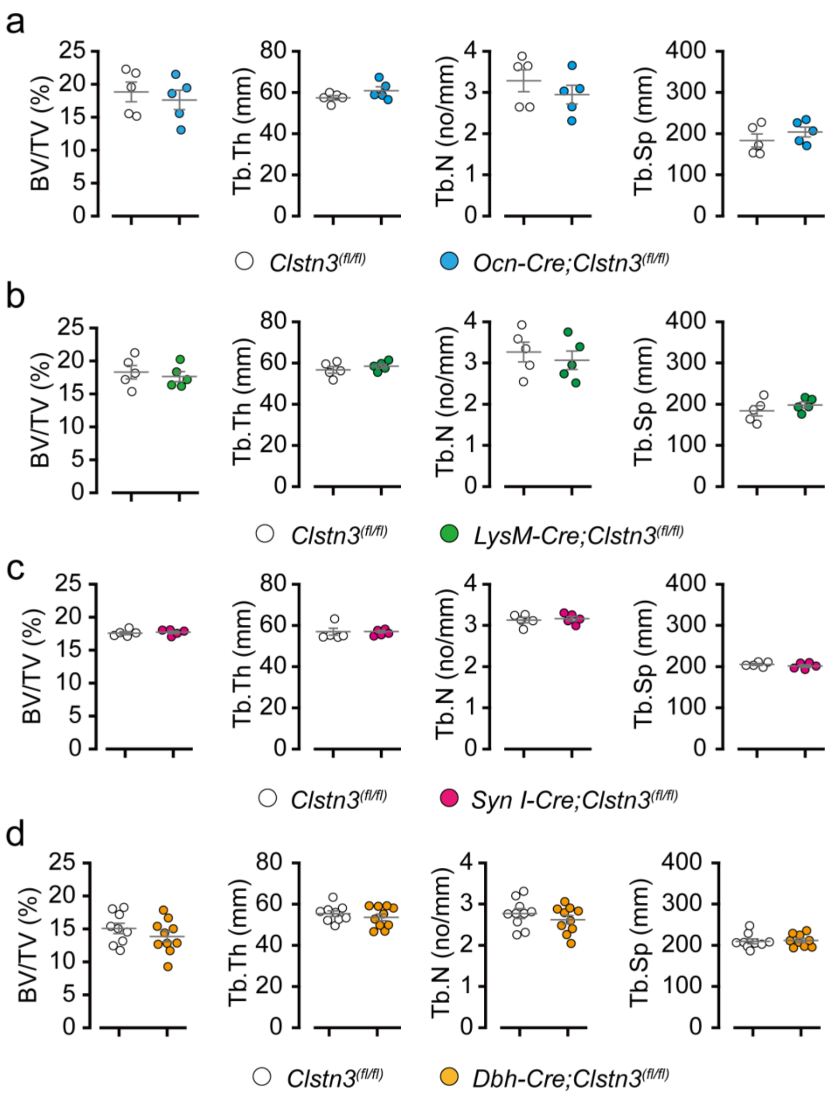

Supplementary Fig. 2 Femural trabecular bone analysis in tissue-specific conditional knockout mice. Quantification of trabecular bone volume fraction (BV/TV), trabecular thickness (Tb.Th), trabecular number (Tb.N), and trabecular separation (Tb.Sp) in **a** osteoblast-specific (*Ocn-Cre;Clstn3*$^{fl/fl}$), **b** osteoclast-specific (*LysM-Cre;Clstn3*$^{fl/fl}$), **c** pan-neuronal (*Syn I-Cre; Clstn3*$^{fl/fl}$), and **d** sympathetic neuron-specific (*Dbh-Cre;Clstn3*$^{fl/fl}$) *Clstn3* knockout mice. Two-sample *t*-tests were performed for statistical analysis.

Supplementary Figure 3

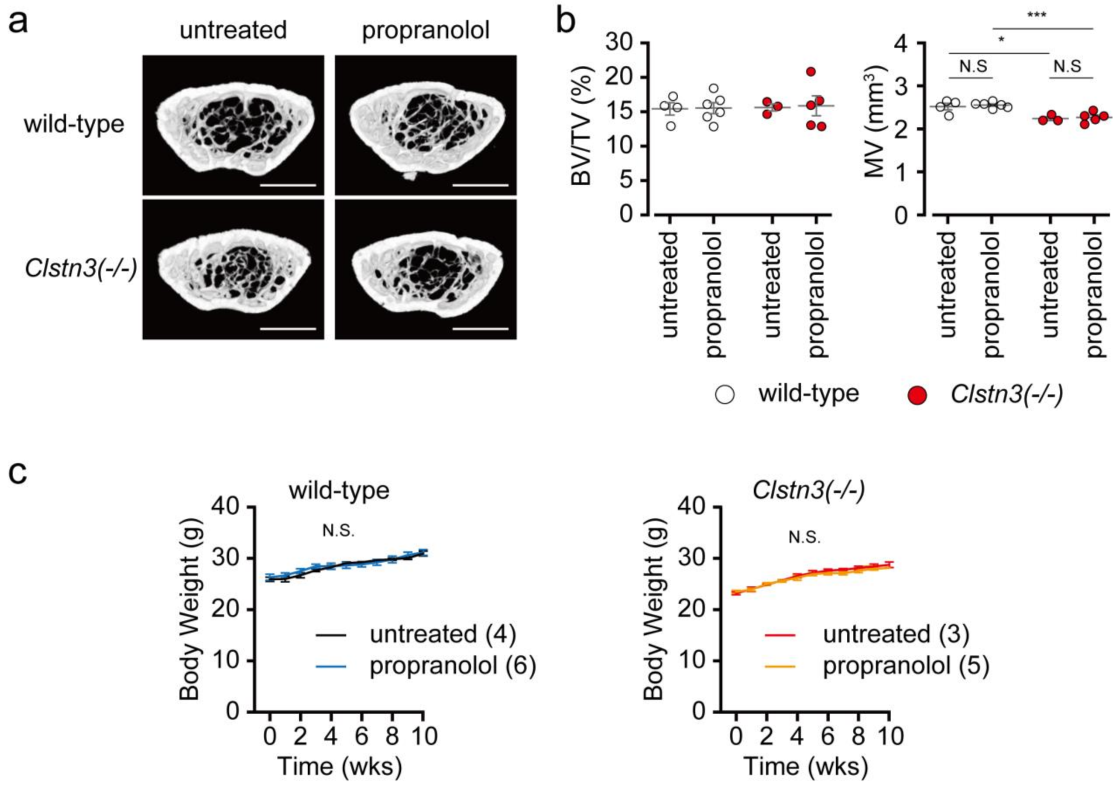

Supplementary Fig. 3 Bone and body weight phenotypes are unaffected by propranolol treatment. **a, b** μCT analysis of untreated or propranolol-treated mice. **a** Representative images of coronal sections in the distal femur metaphysis and **b** a quantification of trabecular bone volume fraction (BV/TV) and marrow volume (MV). Analysis of variance (ANOVA) with post hoc Tukey test were performed for statistical analysis. Scale bar, 1 mm. **c** Body weight of wild-type and *Clstn3* knockout mice treated with propranolol. Two-sample *t*-tests were performed for statistical analysis.

Supplementary Figure 4

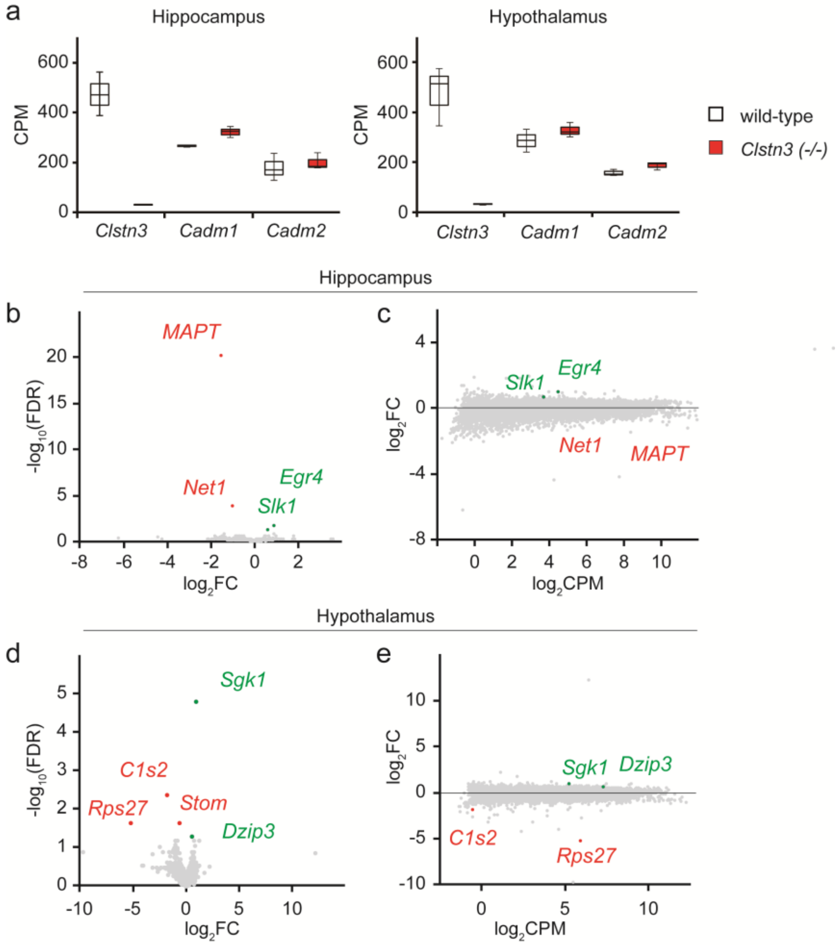

Supplementary Figure 5 Bulk RNA-seq analysis a Boxplots indicating the expression of *Clstn3*, *Cadm1*, and *Cadm2* in wild-type and *Clstn3* global knockout mice (n=3 for each group). **b,d** Volcano plots displaying differentially expressed genes from *Clstn3* global knockout mice compared with wild-type controls in the hippocampus (**b**) and hypothalamus (**d**). **c,e** MA plots displaying differentially expressed genes from *Clstn3* global knockout mice compared with wild-type controls in the hippocampus (**c**) and hypothalamus (**e**). This plot visualizes the differences between two groups by transforming the data onto M (log ratio) and A (average) scales and then plotting these values. Green dots represent up-regulated genes and red dots represent down-regulated genes.

Supplementary Table 1

Supplementary Table. 1 Primers used in this study.

Gene	Sense (5'→3')	Antisense (5'→3')
Clstn3 (conventional)	GCTGCGACCGAGACAACTAT	AGGGCGTAGTGATGCCATTC
Clstn3 (quantitative)	ACCTAGCTGGTTTCAGCGTG	CACGTGGACCTTCATGCCTT
Clstn3b	CTCCGCAGGAACAGCAGCCC	AGGATAACCATAAGCACCAG
Sp7	GTCCTCTCTGCTTGAGGAAGAA	GGCTGAAAGGTCAGCGTATG
Bglap	CTGACAAAGCCTTCATGTCCAA	GCGCCGGAGTCTGTTCACTA
Nfatc1	AGGGCTCACTATGAGACGGA	CAGCTGTAGCGTGAGAGGTT
Ctsk	GAAGAAGACTCACCAGAAGCAG	TCCAGGTTATGGGCAGAGATT
Gapdh	AGGTCGGTGTGAACGGATTTG	TGTAGACCATGTAGTTGAGGTCA
Rn18s	GGCCGTTCTTAGTTGGTGGAGCG	CTGAACGCCACTTGTCCCTC

Supplementary Table 2

Supplementary Table. 2 Synaptic adhesion molecules enriched in the hypothalamus of high fat-fed mice (GSE127056).

Gene symbol	log_2FC	*p*-value
Slitrk1	3.41E-01	0.01837252
Dscaml1	3.08E-01	0.00553559
Cadm1	2.96E-01	0.01918594
Efna5	2.77E-01	0.04405432
Nlgn1	2.33E-01	0.0469376
Nrxn3	2.26E-01	0.04484547
Negr1	2.24E-01	0.02793544
Nlgn3	2.16E-01	0.01553429
Slitrk5	1.84E-01	0.01852295
Ncam1	1.77E-01	0.04914928
Sdc2	1.64E-01	0.03644869
Clstn3	1.37E-01	0.04091735
